# Mental health consequences of parental death and its prevalence in children: A systematic literature review

**DOI:** 10.1016/j.heliyon.2024.e24999

**Published:** 2024-01-19

**Authors:** L.V. Cabal Aguirre, A.K. Jaramillo, T.E. Saucedo Victoria, A. Botero Carvajal

**Affiliations:** School of Health, Universidad Santiago de Cali, Cali, Colombia

**Keywords:** Mental health, Parental death, Prevalence, Consequences, Systematic literature review

## Abstract

The death of a loved one can occur at any stage of life and can have a profound clinical impact on the patient. During childhood and adolescence, smoking has a functional impact on key aspects of family, school, and social life. The negative effects of parental death on children's mental health and its prevalence are unknown. Therefore, this systematic literature review aimed to describe the effects of parental death on children's mental health and its prevalence. The PubMed/Medline, WoS, and Cochrane Trials databases were searched for articles on patients aged 4–18 years. This review examines three articles. Anxiety and depression were identified as the predominant mental health outcomes, with a prevalence ranging from 7.5 % to 44.67 % of the mental health consequences associated with parental death.

## 1 Introduction

**Fig. 1:**
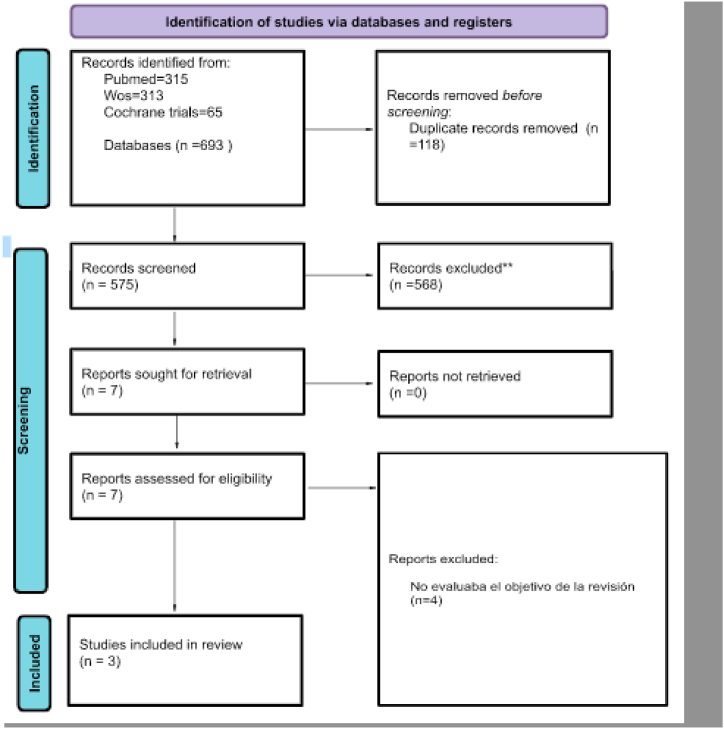
PRISMA flowchart diagram.

**Fig. 2:**
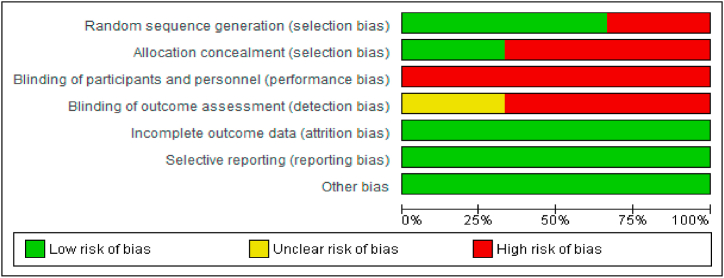
Risk of bias of included studies in general

**Source:** RevMan 5.4

**Fig. 3:**
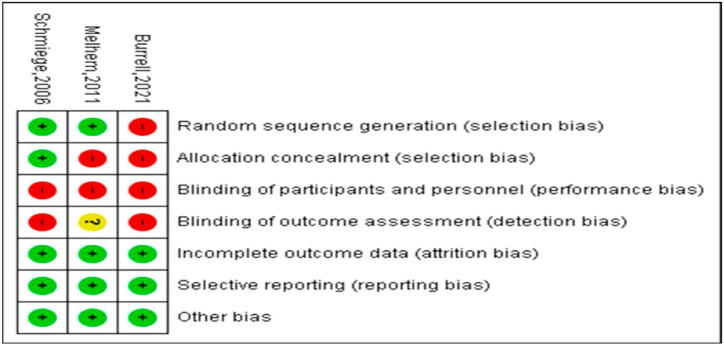
Risk of bias by study and criterion

**Source:** RevMan 5.4

At any stage of the life cycle, death is an inevitable and irreversible event. Typically, there is no prior preparation that enables a better understanding and deployment of coping mechanisms. Therefore, losing a parent is a traumatic life event that causes children and adults to experience a variety of emotional issues. According to statistics, between 3.5 % and 4 % of children worldwide lose a parent [1](Hoyos, 2015). In addition, in the U.S., 4.12 %, or more than 2.9 million children under the age of 18, had previously experienced the passing of their parents (3.47 %) [2] (Burns et al., 2020). Moreover, approximately 1.8 million children in Mexico are not under the care of either their mother or father [3] (Diaz, 2018) (see Fig. 1, Fig. 2, Fig. 3).

Furthermore, parental death triggers a grieving process as an adaptive response, which involves significant changes and increased adaptation since it has an effect on the psychological level [4] (Fonnegra, 2009). In addition, it causes emotional, cognitive, behavioral, and physiological symptoms [5] (Enez cited by Lansford et al., 2021). In this regard, several studies have revealed a variety of mental health issues resulting from parental loss, such as depression, anxiety, PTSD, externalizing and internalizing problems, psychoactive substance use disorder (PAS), and other schizoid, psychotic, and personality symptoms [6](Guillén et al., 2013). However, the health sciences seldom discuss the frequency of mental disorders that result from losing a parent in childhood. The aim of this study was to determine the mental health outcomes of children aged 4–18 years who had not received any treatment after the news of their parent's death. Additionally, the present study aimed to estimate the prevalence of mental health problems in these children.

For this reason, investigating the frequency of psychological disorders associated with parental death in childhood and adolescence is necessary. Currently, there are few studies in the scientific literature that offer statistical data about the frequency of parental death at various stages of the life cycle. This may be related to factors such as the limited number of studies on mourning, the lack of agreement among professionals in the field about diagnostic methods, and the variety of symptoms that arise throughout the mourning experience. All of these factors result in the lack of a thorough analysis of this issue and how prevalent it is in mental health [2].

In addition, according to some studies, there are several effects of parental death on children's and adolescents' mental health [7,8] (Appel et al., 2019; Lutz et al., 2012).

First, there are physical symptoms that alter the functioning of the person and cause significant discomfort.

Second, emotional symptoms of a depressive kind are observed through manifestations such as crying, sadness, and sleep problems. Additionally, anxiety symptoms manifested as separation anxiety and excessive fear. Additionally, there may be behavioral problems expressed through disruptive behaviors, which also affect the learning process and school performance.

Finally, it is important to note that these consequences and the frequency of mental disorders in this population cause clinically significant discomfort, which affects performance and everyday functioning.

## 2 Method

### 2.1 Protocol and registration

This study followed the suggested reporting elements for systematic reviews (PRISMA). (PRÓSPERO, CRD42022329541).

### 2.2 Eligibility criteria and study selection

The following have to meet the requirements in order to qualify (PICOT criteria): (1) participants, children aged between 4 and 18; (2) exposure, parental notice of death; (3) comparison, not applicable; (4) outcomes, mental health; (5) study design, cohort or clinical trial; (6) study design, cohort or clinical trial.

### 2.3 Information sources and search tools

An electronic search of the Scopus, Web of Science, MEDLINE/PubMed, Clinical Trials.gov, and Cochrane Central Register of Controlled Trials databases was also conducted. Controlled trials, cohort and longitudinal studies, and English language articles up to May 3, 2022, with no language or date restrictions were searched.

(“parental death" [MeSH Terms] OR (“parental" [All Fields] AND “death" [All Fields]) OR “parental death" [All Fields]) AND (“mental health" [MeSH Terms] OR (“mental" [All Fields] AND “health" [All Fields]) OR “mental health" [All Fields]) AND (“child" [MeSH Terms] OR “child" [All Fields] OR “children" [All Fields] OR “child s" [All Fields] OR “children s" [All Fields] OR “childrens" [All Fields] OR “childs" [All Fields])

### 2.4 Selection and data collection process

Study titles and abstracts that were found during the searches were evaluated separately by two reviewers. Duplicate and irrelevant titles and abstracts were eliminated. The potential candidate full-text articles were then independently retrieved by the reviewers for additional assessment. All disagreements were resolved through discussion, and if they persisted, a third reviewer was consulted to make the final decision.

The characteristics of the study population included the following: (i) the author's first surname; (ii) the year of publication; (iii) the country; (iv) the study setting; (v) participant characteristics, sample size, and mean age; (vi) exposure characteristics (parental role, type of death); and (vii) hazard ratios with 95 % confidence intervals for mental health outcomes, as determined by an instrument.

Prognosis was measured by an instrument that assesses mental health problems related to the emotional, psychological, and social well-being of individuals or groups. Hazard ratios were calculated to determine the prognosis. Since the present study aimed to determine the prevalence of symptoms rather than a specific diagnosis, our focus was to highlight the importance of symptomatology.

When the necessary information was not obtained, the authors of the article were contacted.

### 2.5 Reporting bias assessment

REMARK is the recommended prognostic study, followed by risk of bias and QUADAS for prevalence studies. Both tests were run via RevMan.

## 3 Results

A search for articles on the prevalence and mental health consequences of parental death in children was also conducted. A total of 693 scientific articles from different countries were obtained. Afterward, 118 duplicates were eliminated, the articles were examined by title and abstract, and all those that did not meet the criteria were thus eliminated. Only seven articles were read in full, but only 3 met the criteria for the present systematic review. These articles were found in the following databases: Pubmed, WoS, and Cochrane Trials. Table 1 shows the sociodemographic data for the 3 publications that were examined. A total of 644 children who had lost a parent between the ages of 4 and 18 participated in the study, and the prevalence of mental health consequences ranged from 7.5 % to 44.67 %. We found that the prognosis for children involves the development of symptoms of anxiety and depression, the prevalence of which ranged from 7.5 % to 44.67 % in the included studies.

**Table 1:** Summary of prevalence and mental health consequences in children.

Authors/Year/Country/Type of study	Characteristics of the total sample	Characteristics of the group	Psychological symptom collection instruments	Prevalence of psychological symptoms and mental health consequences	Mental health variables collected
Schmiege. 2006/Arizona, U.S.A./Randomized controlled trial, longitudinal approach Growth curve methodology	244 participants Type of death: Not applicable Filial relation: Biological parent or parent figure (i.e., someone with a parent role at least 2 years prior to death)	109 children between 8 and 16 years old average of 11.39. Female (n: 52) 47.71 % Male (n:57) 52.29 %	Children's Manifest Anxiety Scale Revised 17 (*R*-CMAS, 28 items); Childhood Depression Inventory (CDI, 27 items); Self-report of youth outsourcing subscales (YSR, 30 items)	Depression: Female: 1st wave (n:52) 47 % 2nd wave (n:50) 48 % 3rd wave (n:48) 49 %. Male: 1st wave (n:57) 52 %, 2nd wave (n:54) 51 %; 3rd wave (n: 49) 50 %. Anxiety: Female: 1st wave (n:52) 47 %; 2nd wave (n:50) 48 %; 3rd wave (n:48) 49 % Male = 1st wave (n:57) 52 %; 2nd wave (n:54) 51 %; 3rd wave (n:49) 50 %. Event studied: 44.67 % Effect size estimates of group differences were 0.46 and 0.36 for girls and 0.02 and 0.12 for boys for anxiety and depression, respectively.	Externalization Problems: 44,67 %
Melhem. 2011/USA/Longitudinal Study	182 participants Type of death: Suicide, unintentional injuries, and sudden natural causes Filial relation: Fathers and mothers	182 children between 7 and 18 years old. Female (n:83) 45.6 % Male (n: 99) 54.40 %	ICG-RC, Inventory of Complicated Grief–Revised for Children version.	Depression: 31.6 % (6 participants F = 21.9 p value < 001) Anxiety: 31.6 % (6 participants F = 18.4 p value < 001) Event studied: 31.6 % (6 participants)	Posttraumatic stress disorder PTSD: 31.6 % Functional deterioration: 31.6 %
Burrell. 2021/Norway/Prospective longitudinal cohort study, between 2008 and 2012	4723 participants Type of death: Suicide, homicide, transportation accident, falls, poisoning or drowning. Filial relation: Father, mother, or both, either biological or adoptive.	353 children between 4 and 18 years old No gender distinction	Central Population Register, International Classification of Deaths Register, Norwegian Specialized Mental Health Services, Statistics Norway's event database, Norwegian Patient Register (NPR),	Depression: 22.38 % (79 participants) HRs with 95 % Cis 2.09 (1.68–2.62)? Anxiety: 9.35 % (33 participants) HRs with 95 % Cis 1.45 (1.03–2.05) Event studied: 7.5 % (353 participants)	SPA consumption disorders: 16.71 % (59 participants) Stress reactions: 14.16 % (50 participants) Personality disorder: 4.53 % (16 participants) Psychotic disorder: 3.12 % (11 participants) Bipolar disorder: 2.83 % (10 participants) Eating disorder: 0.85 % (3 participants)

First, in terms of heterogeneity through random sequence generation (selection bias), the studies by Schmiege et al. (2006) [9] and Melhem (2011) [10] obtained a low risk score (70 %), and those by Burrell (2021) [11] obtained a high risk score (30 %).

Second, in terms of allocation concealment (selection bias), the studies by Burrell (2021) [11] and Melhem (2011) [10] obtained a high risk score (70 %), and those by Schmiege et al. (2006) [9] obtained a low risk score (30 %).

Third, due to the type of circumstance being examined, it was not possible to blind the participants in the three studies included in the review [[9], [10], [11]]. Therefore, in terms of developmental bias, three studies obtained a high risk score (100 %).

Fourth, in terms of selection bias, the study by Melhem (2011) obtained an unclear risk score (30 %), and those by Schmiege (2006) and Burrell (2021) obtained a high risk score (70 %).

Finally, in three of those studies [[9], [10], [11]], a low risk of attrition, selection, or other biases was evident, corresponding to 100 % of these categories.

## 4 Discussion

The results of the analysis indicate that depression and anxiety are the most common mental health consequences associated with parental death, with a prevalence rate between 7.5 % and 44.67 % [[9], [10], [11]]. This result is consistent with the results from previous studies on mental health consequences and the prevalence of psychological disorders [12](Böckerman et al., 2022). This similarity occurs because depressive symptoms experienced during a period of grief are an adaptive response to loss. However, when such symptomatology persists over time, it can lead to outcomes that are clinically significant in childhood, adolescence, and early adulthood [6,7,13,14].

Major depression accounts for 15.9 % [15] of moderate symptoms as a response to grief, along with other consequences, such as social isolation, emotional blockage, depression, and anxiety [16].

Therefore, there may be three primary causes for the mental health consequences linked to parental death. First, the affected person experiences social isolation, which is related to the depression phase that is a natural part of the mourning process. Second, the taboo on death in certain cultures makes it difficult to discuss the issue at home and in classrooms. This is especially evident when discussing death with children, as they lack the understanding required to deal with the event at such a young age. Third, according to Guillem et al. [17] certain causes of death, particularly suicide or violent crimes, are socially stigmatized.

The mental health consequences derived from the loss of a parent increase the risk that aggression, inappropriate sexual behavior, and drug misuse may occur. In addition, education affects the educational process because it significantly influences the mood of the mourner at school and may lead to issues regarding identity and self-esteem that reduce academic performance and classroom interaction [18,19].

Other studies on the subject support the notion that the effects of grief experienced throughout adolescence have mental and physical consequences, decrease quality of life, and interfere in various areas, such as the academic and social spheres. In the school setting, performance declines, and dropout rates increase. In the social setting, this approach increases the likelihood of generating risky behaviors that continue into adulthood [20,21].

According to the results of the aforementioned authors, losing a parent is a stressor event that triggers grief and significantly affects the triple response system, that is, the emotional, cognitive, and behavioral levels. In turn, these components connect to each of the vital spheres—that is, personal, family, school, and social life—in which an individual grows. Therefore, the dynamics of each sphere may shift negatively as a result of parental loss [22].

According to Melhem [10] losing a parent can lead to functional impairment, which negatively affects personal, social, family, school, and work functioning. This results in the development of psychological symptoms, the progression of those symptoms, and the presence of psychopathologies. According to diagnostic manuals for mental disorders, such as the International Classification of Diseases and Related Health Problems (ICD-10) and the Diagnostic and Statistical Manual of Mental Disorders (DSM-5), functional impairment is linked to the factors of frequency, intensity, and duration of symptomatology; this allows for the determination of the level of exacerbation and if the individual presents clinically significant discomfort and functional impairment indicative of a psychopathology [23,24].

Moreover, some studies show consequences related to the lack of protection. According to these studies, the loss of a parent exposes the mourner to social stigmatization [25]. In addition, they reported a decline in their psychosocial functioning, which raises the possibility that they may struggle with their self-esteem and their degree of satisfaction with life [26].

Therefore, studies suggest that psychosocial problems [10] and low self-esteem have negative effects [9]. In this context, young children who lose a parent, or to a greater extent, both parents, experience a lack of protection and a decline in their psychosocial well-being. This negatively impacts the self-esteem and level of life satisfaction of children and adolescents because it reduces the extent to which physiological, security, belonging, self-esteem, and recognition needs are met and satisfied, interfering with their ability to experience self-fulfillment, a need that is satisfied with achievements at later stages [26].

In addition, because suicide is typically viewed as a repulsive act and is thus forbidden to discuss in public due to the numerous allegations that may be made against it, it creates an enormous emotional load and hinders the grieving process for the family that is exposed to the loss [27,28]. This is directly related to the prevalence of psychological disorders since, as was discovered in one of the studies included in the present study, parental suicide is clearly associated with a greater probability of experiencing any type of mental disorder in the years following loss [11].

Another finding in the current study was posttraumatic stress disorder (PTSD), which has a prevalence rate of 31.6 % [10]. In this context, studies by Melhem and Brent [15] and Zhou H et al. (2016) [29] indicate prevalence rates of 15.9 % and 72.6 %, respectively, in response to grief. PTSD is highly prevalent, followed by psychological disorders, such as depression and anxiety, because death can occur suddenly and unexpectedly due to causes such as suicide, natural causes, violent acts, accidents, natural disasters, or multiple losses, which can complicate the grieving process. After all, this practice intensifies the frequency, intensity, and duration of the symptomatology [30].

Other consequences found in this study include externalizing symptoms, for which the prevalence rate was 44.67 % in childhood and adolescence [9]. In this context, experiencing parental death results in significant behavioral changes since children and adolescents have not yet fully developed their cognitive and behavioral emotional resources or coping mechanisms, allowing them to deal with such losses more effectively and reducing the possibility of developing maladaptive behaviors [23,31].

In addition, according to some studies, parental loss can cause external and internal issues [32]. According to this study, external difficulties are more prevalent in women, and internal difficulties are more prevalent in men [9].

This is related to social and cultural factors because, according to cultural and social traditions, men should assume power-oriented roles, which means a high level of emotional control and few opportunities for expressing such emotions. In contrast, women tend to play greater nurturing roles, which affects how quickly they can recognize and express their emotions. This explains why, in the face of a stressful life event, such as the death of parents, there is a greater tendency for men to express their emotions through maladaptive behavioral reactions, although there is evidence that this is not the case for women [14,33,34].

The prevalence of stress reactions, which was 14.16 % in this study's analysis of the mental health consequences of parental death, is another important finding [11] (Burrell, 2021). These reactions are associated with the triple response system because experiencing this event at a young age can overpower an individual's limited coping mechanisms and adaptive capabilities, leading to significant manifestations of stress that may be physical, emotional, cognitive, or behavioral and significantly affect their overall functionality [22] (Kentor & Kaplow, 2020).

In addition, Burrell (2021) [11] reported a prevalence of 16.71 % for PAS. This is connected mainly to the adolescent stage because this period is characterized by significant changes in the body, brain, interpersonal relationships with peers and family members, and individual autonomy. This makes them more vulnerable to developing risky behaviors that are maladaptive when they face stressful life events [5,34].

In this context, according to a study conducted at the turn of the century, individuals who experienced various adverse childhood experiences, such as grieving during childhood and adolescence due to the loss of parents, were 4–12 times more likely to experience problems with alcohol consumption, use of psychoactive substances, and mood disorders such as depression. Similarly, the risk of experiencing problems, such as smoking and sexually transmitted diseases, increases two to fourfold [20].

The prevalence rates of other disorders found in this study were as follows: psychotic disorders, 3.12 %; bipolar disorder, 2.83 %; and eating disorders, 0.85 %. These disorders are the least prevalent [11].

According to the literature review, parental death is suggested to result in adverse mental health outcomes in children exposed to the death of a parent. However, we emphasize the importance of considering that adverse effects are greater when parents and children share the same sex. Therefore, it is crucial to consider the effect of sex in future analyses when designing postparental crisis interventions or care programs and during follow-up over time [12].

In conclusion, depressive and anxiety disorders are the most common consequences of parental death during childhood and adolescence, followed by PTSD and other external problems. However, the prevalence of PAS consumption disorders and stress reactions has also been highlighted. In addition, other less prevalent disorders, such as personality disorders and eating disorders, are noted. Therefore, all these consequences are attributable to loss due to parental death during childhood or adolescence and are the cause of significant functional impairments in vital areas of the individual.

Losing a parent can have a significant impact on a child's mental health. To help affected children, policymakers should consider providing grief counseling services in schools and communities, as well as easy access to information and services related to mental health. Legislators can advocate for comprehensive support systems, such as counseling, educational materials, and community activities, to promote children's mental well-being during this difficult time.

## 5 Limitations

The studies used were published in English, as it is the universal language used in scientific research.

Studies in other languages and in other databases in Spanish were not included.

Our study has several strengths. The NRS-2002 provides appropriate evidence for patient prognosis: cohort studies and randomized or nonrandomized clinical trials. The prevalence of NAFLD was reported through the inclusion of observational and cohort studies. To the best of our knowledge, this is the first review summarizing the prognosis for mental health following parental death in individuals aged 4 to 18 and reporting its prevalence. The bias assessment suggested that the included studies collectively had a low level of bias; therefore, the recommendations derived from the study for mental health professionals to provide psychological support for parental loss are advisable. The databases included in the study allowed us to locate articles more closely aligned with the criteria outlined in the registered protocol.

## Funding details

This research was funded by Dirección General de Investigaciones of Universidad Santiago de Cali under call No. 02-2023.

## Data availability statement

The data can be found in the manuscript or by request to the corresponding author.

## CRediT authorship contribution statement

**L.V. Cabal Aguirre:** Writing – review & editing, Writing – original draft, Validation, Formal analysis, Data curation, Conceptualization. **A.K. Jaramillo:** Writing – review & editing, Writing – original draft, Formal analysis, Data curation. **T.E. Saucedo Victoria:** Writing – review & editing, Writing – original draft, Validation, Data curation, Conceptualization. **A. Botero Carvajal:** Writing – review & editing, Writing – original draft, Supervision, Project administration, Funding acquisition, Conceptualization.

## Declaration of competing interest

The authors declare the following financial interests/personal relationships which may be considered as potential competing interests:ALEJANDRO BOTERO CARVAJAL reports financial support and article publishing charges were provided by Santiago University of Cali. ALEJANDRO BOTERO CARVAJAL reports a relationship with Santiago University of Cali that includes: employment and funding grants.
